# Hidden culprit of suprascapular neuropathy: independent suprascapular notch entrapment coexisting with spinoglenoid notch cyst - a case report

**DOI:** 10.1016/j.xrrt.2026.100879

**Published:** 2026-09-07

**Authors:** Georges Sakhat, Hady Ezzeddine, Mohamad Badra, Ramzi Moucharafieh, Mohamad El Dassouki, Mohamad Yazbeck, Mohammad Nedal Jomaa

**Affiliations:** a Department of Orthopaedic Surgery and Traumatology, University of Balamand, Beirut, Lebanon; b Department of Orthopedic Surgery, Clemenceau Medical Center, Beirut, Lebanon; c Department of Neurology, Lebanese American University School of Medicine, Lebanon; d Department of Neurosurgery, Clemenceau Medical Center, Beirut, Lebanon

**Keywords:** Suprascapular neuropathy, Entrapment neuropathy, Spinoglenoid cyst, SLAP tear, Transverse scapular ligament, Suprascapular notch, Denervation edema

## Introduction

Suprascapular neuropathy (SSNeur) is a well-recognized cause of shoulder pain and weakness. It is most often caused by either compressive lesions such as cysts or tumors,^3^^,^^4^^,^^18^ or by traction injuries from repetitive overhead activities (highly prevalent in volleyball players) or from rotator cuff tear retraction.^2^^,^^3^ The 2 classical sites of compression are the suprascapular notch (SSNo) and the spinoglenoid notch (SGNo).^19^ These 2 notches represent narrow and bent passageways where the nerve gets tethered to the scapula at an angle, limiting the nerve's mobility. Although compression at one notch is the usual scenario, extension of the compressing etiology from one notch to the other has been sporadically described, creating what has been termed *dual-notch* entrapment.^7^^,^^11^^,^^20^

We present the case of a 56-year-old male patient with left shoulder weakness who was found to have suprascapular nerve (SSN) compression at the SSNo by the transverse scapular ligament (TSL) and a concomitant small paralabral cyst confined to the SGNo. Prior reports have described dual-site compression by a single lesion including intraneural ganglion cysts^11^ and large spinoglenoid cysts (SGCs).^10^^,^^16^ But to the best of our knowledge, *simultaneous* bifocal entrapment by 2 *separate* and *independent* lesions has never been described before. The current report is the first surgically documented case of dual-site SSN compression caused by 2 different mechanisms/lesions. This report underscores the need for careful preoperative clinical reasoning when the presence of a SGC does not adequately account for the pattern or severity of SSNeur demonstrated on examination, electrodiagnostic studies, and imaging. Specifically, the involvement of the supraspinatus on clinical and paraclinical tests predicts concomitant SSNo compression.^3^^,^^4^^,^^11^^,^^19^^,^^20^

## Case presentation

The patient is a 56-year-old, right-hand dominant male who works as a media professional for an international organization. At the time of presentation, he was serving his duties in Lebanon. He was lean, healthy, with no self-reported history of diabetes or endocrinopathies, and he engaged in calisthenics and swimming as leisure activities. He presented to our shoulder clinic in February 2025 with marked left shoulder weakness and visible scapular muscle atrophy (Fig. 1).

**Figure 1:**
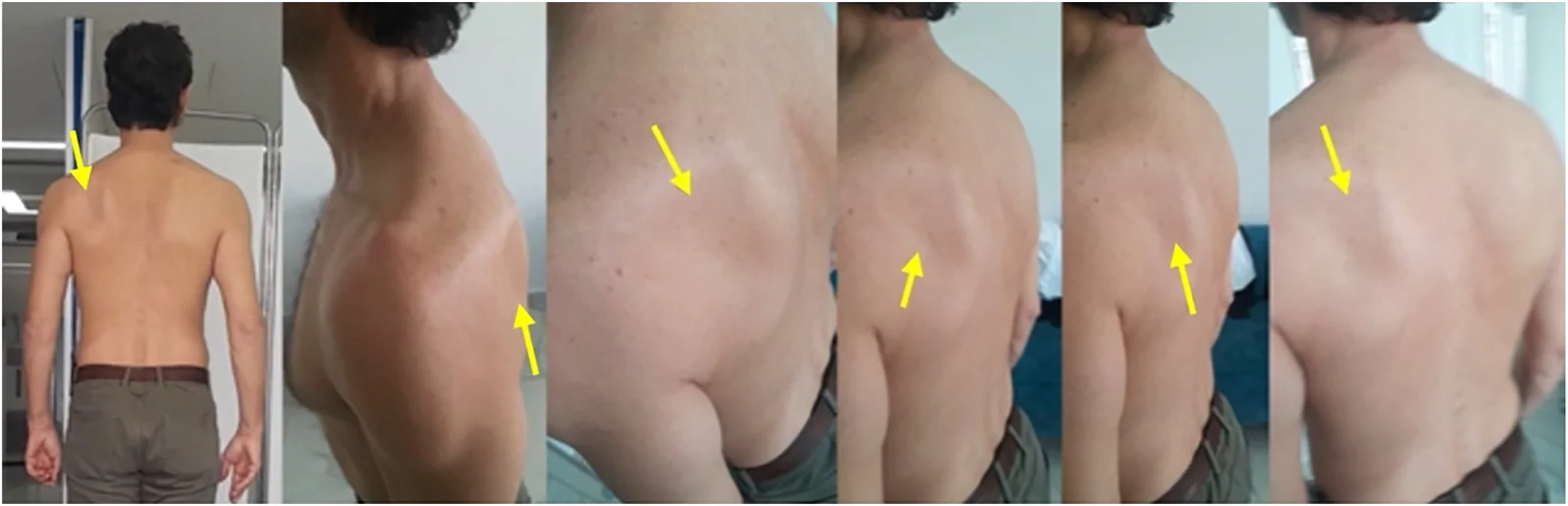
Clinical photographs showing marked infraspinatus atrophy on the left side (*yellow arrow*).

He attributed symptom onset to a prior meningioma excision performed in December 2023 in another country. He reported that his left shoulder weakness began immediately upon awakening in the intensive care unit after that surgery. He denied any prior shoulder symptoms. Apart from the shoulder condition, the meningioma surgery was successful. His most recent brain magnetic resonance imaging (MRI), performed one week before presentation, was normal. The patient was unable to retrieve his operative report or medical file from the hospital abroad.

During postoperative hospitalization, he experienced intense nonradiating burning pain in the posterolateral aspect of the left arm and was unable to elevate the arm from the moment he woke up from surgery. He described a “paralyzed” arm. He was discharged with no clear diagnosis on conservative treatment, including pregabalin and physiotherapy. The pain was not excruciating, overwhelming, or diffuse in character. It lasted for approximately two weeks, then localized to the scapular region before resolving over several weeks. Since then, he reported no continuous pain or sensory deficits, but he described infrequent sudden, spontaneous and brief electrical “stabs” that forced him to wiggle and shake the limb in the dependent position to alleviate these stabs. The patient had one of these stab episodes during our physical examination and we watched him wiggle his limb as he described (Supplementary Video S1).

Although his perceived range of motion (ROM) recovered almost completely shortly after onset, and although the paralysis had disappeared, his shoulder strength hadn't recovered and it continued to decline. He first noticed weakness during gym workouts and later during work and activities of daily living. Approximately seven months after surgery, he noticed muscle wasting over the scapula. He denied instability or restriction of motion. The symptoms were limited to infrequent dysesthesia and persistent weakness and atrophy refractory to one year of strengthening physiotherapy.

Examination revealed noticeable infraspinatus wasting (Fig. 1). There was no scapulothoracic winging or abnormal motion on either side. Neck ROM was normal and unlimited, and it did not elicit any pain. Active ROM of both shoulders was almost full and is shown in Table I and in Supplementary Video S1. The patient was unaware of the terminal 20° deficit in active ER1. Passive ROM of the left shoulder was not limited and was symmetric to the right shoulder. Left supraspinatus provocative testing was weak but nonpainful. There were no signs of tear, tendinitis, or subacromial impingement. Left Bear Hug, Belly Press, and Gerber's lift-off tests were normal. Left side external rotation in ER1 position was limited and weak (20° deficit, MMT grade 3−/5) while external rotation strength in the ER2 position was relatively preserved (4+/5). The Hornblower test and sign were negative. Anterior apprehension test was negative for anterior instability. Kim and Jerk tests were negative for posterior instability. O'Brien test was positive. The acromioclavicular joint was stable and nontender. The Lafosse suprascapular stretch test (contralateral cervical rotation with scapular retraction) was positive on the left and negative on the right. It elicited a deep, dull posterolateral pain “similar to his electric stabs”. Spurling test (cervical extension, ipsilateral rotation and bending, and axial compression) was negative on the left. It didn't elicit the pain that was experienced with the suprascapular stretch test. The anterior, middle and posterior heads of deltoid had strong palpable contractions, MMT 5/5, bilaterally. They seemed to the senior author and operating surgeon (M.J.) to be stronger on the affected than nonaffected side (compensatory phenomenon). The biceps contracted vigorously and was symmetrical to the right side. Rhomboid testing showed palpable strong contraction during resisted scapular retraction. Serratus anterior testing showed strong scapular protraction and excellent scapular stability against the ribcage on resisted forward elevation. Both trapezius muscles had a palpable strong and symmetric contraction. Scapulothoracic and glenohumeral motions were harmonious. The pectoralis major and latissimus dorsi were strong and appropriately engaging through the ROM.

**Table I:** Table showing the preoperative active range of motion, passive range of motion, and strength of both shoulders.

	Right shoulder (asymptomatic)			Left shoulder (affected)		
ROM direction	Active ROM	Passive ROM	Strength (pounds)	Active	Passive	Strength (pounds)
Forward elevation	180°	180°	-	180°	180°	-
Lateral elevation	180°	180°	22	180°	180°	17
ER1	80°	80°	15	60°	80°	3
ER2	90°	90°	-	90°	90°	-
IR1	T6	T6	-	T5	T5	-
IR2	80°	80°	-	80°	80°	-

ER1: external rotation while the arm is adducted at the side with the elbow flexed to 90°. ER2: external rotation while the shoulder is abducted to 90° with the elbow flexed to 90°. IR1: internal rotation with the arm adducted at the side with the hand (thumb up) trying to reach out the highest vertebral level behind the back. IR2: internal rotation with the shoulder abducted to 90° with the elbow flexed to 90°.

Isometric strength testing was conducted using a dynamometer for external rotation strength (ER1 position) and lateral elevation strength (with the arm held at 90° of abduction in the scapular plane and the hand pronated facing the ground).

*ER,* external rotation; *IR,* internal rotation; *ROM,* range of motion.

Isometric strength testing using a handheld dynamometer (single trial measurement, peak strength recorded) showed external rotation strength of 3 lbs. on the left vs. 15 lbs. on the right (80% reduction). Lateral elevation strength was 17 lbs. on the left vs. 22 lbs. on the right (23% reduction). The main physical examination findings are shown in Supplementary Video S1.

Magnetic resonance arthrogram conducted in February of 2025 ruled out rotator cuff tears. It revealed a well-circumscribed lobulated fluid-filled cystic lesion filling the SGNo and measuring 1.3 × 0.8 × 0.7 cm (Fig. 2). It was contiguous with the posterosuperior labrum but without evident labral tear. Mild atrophy (according to the Warner classification) and denervation edema of the infraspinatus muscle were noted, especially in the most medial fibers. There were mild atrophy and mild edema of the supraspinatus muscle. There was no fatty infiltration of either the supraspinatus or infraspinatus. Teres minor and posterior deltoid hypertrophy were noted. The visible portions of all other muscles including the deltoid, biceps, rhomboids, and serratus anterior showed no atrophy or denervation changes as compared to the contralateral side.

**Figure 2:**
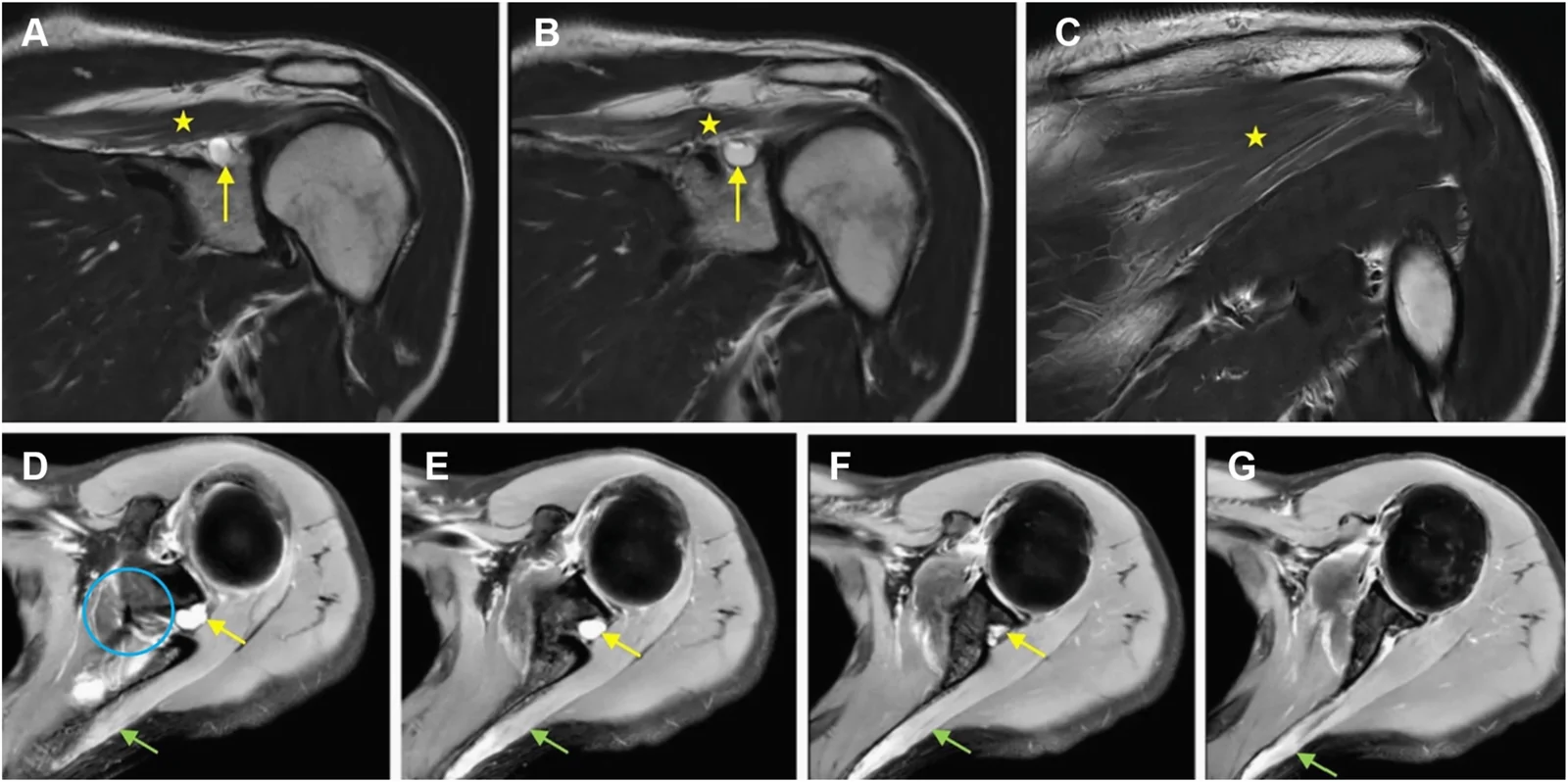
Magnetic resonance arthrogram of the left shoulder. (**A** and **B**) Coronal oblique T2-weighted PROPELLER (Periodically Rotated Overlapping Parallel Lines with Enhanced Reconstruction) images revealing a well-circumscribed, lobulated, fluid-filled cystic structure within the spinoglenoid notch, contiguous with the posterosuperior glenoid labrum—consistent with a paralabral ganglion cyst (*yellow arrow*). Also note the atrophy of the supraspinatus muscle (*yellow star*) and its diffuse T2 hyperintensity relative to the deltoid and subscapularis. (**C**) Coronal oblique T2-weighted PROPELLER image showing diffuse high T2 signal of the infraspinatus (*green star*) compared to the deltoid, teres major and minor. (**D-G**) Axial proton density–weighted, fast spin echo, fat-suppressed, PROPELLER images showing a spinoglenoid notch cyst contiguous with the posterosuperior glenoid labrum. The images also show decreased muscle bulk of the infraspinatus muscle with diffuse hyperintensity that is most prominent in its most medial fibers (*green arrow*). The posterior deltoid appears relatively hypertrophied, suggestive of compensatory muscular adaptation. Supraspinatus and infraspinatus muscle hyperintensity in Figure is consistent with denervation muscle edema. Note in Figure 2*D* the absence of extension of the cyst into the TSL (*blue circle*) at the medial aspect of the base of coracoid. The suprascapular canal at the supraspinatus fosse is spared from any lesions as well. *TSL*, transverse scapular ligament.

Nerve conduction studies (NCS) performed in February of 2025 using Erb's point stimulation (Table II) demonstrated a side-to-side severely delayed distal motor latency into the left supraspinatus (+3.2 ms relative to the contralateral side) and left infraspinatus (+3.4 ms). They showed also a side-to-side severely reduced compound muscle action potential for the supraspinatus (−94%) and for the infraspinatus (−99.5%). Interestingly, the difference in distal latency between the affected supraspinatus and the affected infraspinatus was 0.6 ms, which was within the normal limit (<1.6 ms) and comparable to the other asymptomatic side (0.4 ms). Thus, the NCS strongly favors a pathology proximal to the innervation of supraspinatus and shows negligeable conduction block between supraspinatus and infraspinatus. NCS study of bilateral median, ulnar, radial, medial antebrachial cutaneous, and axillary nerves were normal.

**Table II:** Nerve conduction studies using Erb's point stimulation and recording from the deltoid, supraspinatus, and infraspinatus muscles.

Muscle (Erb point stimulation)	Parameter	Right (asymptomatic)	Left (affected)	ULN (for latency) and LLN (for amplitude)∗	Side-to-side difference	ULN (for latency) and LLN (for amplitude)∗
Deltoid	Latency	3.5 ms	4.0 ms	5.4 ms	+0.5 ms	0.5 ms
	Amplitude	9.7 mV	11.2 mV	4.6 mV	+15.5%	54%
Supraspinatus	Latency	2.8 ms	6.0 ms	4.3 ms	+3.2 ms	0.7 ms
	Amplitude	8.4 mV	0.5 mV	1.6 mV	−94.0%	48%
Infraspinatus	Latency	3.2 ms	6.6 ms	4.8 ms	+3.4 ms	0.4 ms
	Amplitude	20.0 mV	0.1 mV	1.5 mV	−99.5%	48%

*ULN*, upper limit of normal (97th percentile); *LLN*, lower limit of normal (third percentile).

∗ ULN and LLN were determined using the surface electrode study of Buschbacher et al^5^ on 100 asymptomatic volunteers. According to this study, the mean difference in distal latency between the ipsilateral supraspinatus and infraspinatus was 0.4 ± 0.5 ms (ULN: 1.6 ms).

Concentric needle electromyogram (EMG) of the left supraspinatus and infraspinatus muscles showed chronic neurogenic motor unit changes, with high-amplitude and prolonged-duration motor unit potentials. Recruitment was poor and spontaneous activity in the form of fibrillation potentials was recorded in both muscles. The EMG study of bilateral deltoids, biceps, triceps, brachioradialis, and first dorsal interossei muscles were within the normal limits.

Ultrasound-guided aspiration was attempted at our institution but was unsuccessful at visualizing the cyst, likely due to the cyst's small size and deep location. No sonographic abnormalities were identified at or proximal to the SSNo.

The working diagnosis was a mechanical compression at the SGNo caused by a paralabral cyst, likely related to a probable SLAP tear which wasn't evident on imaging. However, the small cyst size (1.3 cm), the absence of its superior extension into the SSNo, and more importantly the electromyographic involvement of the supraspinatus suggested an additional proximal compression of the nerve at the SSNo. A decision to explore the SSNo was thus made *before* surgery.

The patient was counseled that partial strength recovery and functional improvement were expected, but complete reversal of chronic atrophy and restoration of symmetric strength might not occur.

### Surgical technique

Surgery was performed at Clemenceau Medical Center, Beirut, in March of 2025. Under general anesthesia with preoperative interscalene block, the patient was placed in the beach-chair position, and portals were developed sequentially as required. Diagnostic arthroscopy with a 30° scope confirmed a posterosuperior labral tear (SLAP type II). The cyst was not decompressed from the articular side and the labrum at the tear site was not elevated at that stage. The reason for that was the senior author's tactical preference in this particular case to not deflate the cyst from intra-articular, but to keep the cyst bulging so it can be identified and accessed subacromially.

The scope was moved to the subacromial space. There were no bursitis or rotator cuff tears. Following thorough bursectomy and limited acromioplasty (enough for maneuverability of the scope and instruments), the scope was shifted to the lateral portal. The leading edge of the supraspinatus was dissected until reaching the base of coracoid. The conoid ligament was identified. After radiofrequency ablation and shaver dissection of fat and areolar tissue, with posterior retraction of the supraspinatus muscle and suprascapular artery, the TSL was visualized. Its fibers appeared degenerated, thickened, and partially calcified, lacking the normal shiny appearance. The SSN was visualized proximal and distal to the ligament. The supraspinatus motor branch appeared to arise directly distal to the TSL. Proximally, the nerve appeared healthy, but at the ligament it was severely compressed, flattened and hyperemic. A switching stick was used to retract and protect the nerve medially. A Kerrison rongeur was employed to excise the partially calcified ligament fibers piecemeal. After complete release, the nerve demonstrated a long hourglass constriction, and it rebounded from the SSNo.

Attention was then directed to the scapular spine. After clearing bursal and fatty tissue, the raphe between supraspinatus and infraspinatus was identified at the spine base. Two switching sticks facilitated a blunt muscle split between supraspinatus and infraspinatus. A small intermuscular interval was developed with careful radiofrequency. The nerve at the SGNo was identified and protected. Unlike at the SSNo, there was no hyperemia, flattening, or focal constrictions. A small yellowish cyst was visualized and was abutting perineural fat, barely if not touching the nerve. We excised it piecemeal with a pituitary rongeur, releasing yellowish fluid into the arthroscopy fluid.

The scope was then returned to the intra-articular space. A labral elevator was used to elevate the unstable portion of the posterosuperior labral defect responsible for the paralabral cyst communication (at 1 o'clock- 2 o'clock), which was then repaired using 2 1.8 mm knotless all-suture anchors. Repair was tailored to restore the seal while avoiding unnecessary superior or anterior labral tightening. No fluid release was observed during labral elevation. The diseased degenerative biceps anchor was tenotomized at its insertion and tenodesed high in the groove. Key operative steps and findings are shown in Supplementary Video S2. For comparative purposes, we have added a supplementary video of a normally appearing SSN in another patient with no clinical or electrophysiological SSNeur (Supplementary Video).

Postoperatively, the patient wore an abduction brace for four weeks to protect the labral repair and tenodesis. Passive ROM began in week one. SSN mobilization was prioritized since day one, with progressive strengthening of supraspinatus and infraspinatus. Submaximal pain-free isometrics of supraspinatus and infraspinatus (neutral position) were initiated immediately. Active-assisted ROM started at week four, with active ROM and full rotator cuff strengthening (especially external rotation) at week 6. Biceps strengthening commenced at week 12. The patient was instructed to perform daily home exercises, including ER1 external rotation using high-resistance TheraBand, progressively increasing the band resistance monthly.

### Follow-up

The patient reported “a subjective increase in shoulder strength” on postoperative day 10. External rotation strength of the operated shoulder, measured with the same handheld dynamometer used preoperatively and by the same examiner as preoperative, was 10 lbs. (compared with 3 lbs. preoperatively and 15 lbs. contralaterally). MMT strength was 4−/5.

At four weeks, the patient relocated to a new country, but regular follow-up was maintained via video communication. At three months, he demonstrated full active ROM, with ER1 equal to the contralateral side. The compensatory ER drift sign shown in Supplementary Video S1 had disappeared. The patient was asked to fill out a complete DASH questionnaire preoperatively, at 8 months and at 14 months postoperatively. The DASH score decreased from 35 preoperatively to 3 at 8 months. Improvement was maintained at 14 months (DASH score of 4).

At 14 months, the patient was satisfied and reported full ROM and strength of the left shoulder. The videos he submitted demonstrated symmetric strength relative to the contralateral side and restored endurance in abduction-external rotation (Supplementary Video S3).

## Discussion

This case documents SSN entrapment at both notches by 2 independent lesions. After we observed the suffering appearance of the nerve at the SSNo and its less pathological appearance at the SGNo, and after reflecting to the preoperative EMG/NCS findings, we consider that probably the most critical retrospective factor leading to a successful outcome was the preoperative decision to explore the SSNo. Without this decision, the proximal entrapment might have remained untreated, probably resulting in a double-crush syndrome.

To our knowledge, this is the first report describing deliberate SSNo exploration in the setting of a small paralabral cyst confined to the SGNo. Importantly, SSNo entrapment is not an entity discovered incidentally during routine arthroscopy. The notch lies medially deep, and its exposure requires deliberate, time-consuming dissection through a submuscular hypervascular fatty connective and bursal tissue that swells with arthroscopy fluid. If a decision to explore this notch is not made preoperatively, no intraoperative clues will lead the surgeon to do it during surgery. The essential lesion at the SSNo was found to be an inflamed nerve severely entrapped by a thickened and partially *calcified* ligament. This differs macroscopically from ligament *ossification*, which was not present in our case, and which has been shown to be related to aging without precipitating SSN entrapment.^8^ The significance of partial calcification in our case is unknown due to the lack of histopathological study, but it can be speculated to be related to chronic local inflammation.

The profound preoperative reduction in suprascapular CMAP amplitudes and chronic neurogenic changes indicated severe motor nerve dysfunction. However, the absence of fatty infiltration and the early burst of strength recovery following decompression argue against extensive irreversible axonal degeneration. The 3-to-10 lb. improvement within 10 days was likely inconsistent with axonal regeneration and suggested that at least a substantial component of the preoperative electrophysiological deficit was a conduction block or nerve ischemia that was functionally reversible, rather than representing complete irreversible axonal loss. There is an even more interesting question: whether the dual-notch compression could have produced a synergistic “double-crush” phenomenon with profound combined conduction failure, while still preserving enough axon continuity for very rapid recovery after decompression. That would make the case electrophysiologically quite unusual and would change the prognostic implications of EMG/NCS findings. We do not think that postoperative analgesia was responsible for strength recovery because preoperative weakness was not associated with pain. Outside the infrequent episodes of “electric stabs”, the patient was completely painless. During preoperative dynamometry testing, the patient was asked whether he felt any pain while he was pushing and he explicitly denied pain. Of note, the rapid restoration of external rotation strength after suprascapular decompression has been published before. Mall et al^17^ followed up 29 shoulders with isolated SSN compression at the SGNo that underwent open decompression. Seventy-two percent had visible/palpable infraspinatus atrophy. Twenty-three of 29 shoulders (79%) demonstrated improved ER strength at only 1 week after decompression. And in the subgroup with ganglion cyst, all regained full ER strength at a mean of 6 weeks (range 1-16 weeks).

Dual entrapment was suspected before surgery because of supraspinatus involvement by the denervation process. According to our judgment, the 1.3 cm cyst could not have extended proximally enough to compress the supraspinatus motor branch(es). This was confirmed intraoperatively, as the cyst was confined to the SGNo, abutting only the perineural fat of the infraspinatus branch without significant nerve compression. There was a long distance between the cyst and the supraspinatus motor branch, as perceived from the subacromial space (Supplementary Video S2). On EMG/NCS, both the supraspinatus and infraspinatus show major latency prolongation; the supraspinatus–infraspinatus differential remains small; and both muscles have profound CMAP reduction. When these findings are integrated with the thickened/partially calcified TSL and severe nerve entrapment, there, the proximal lesion becomes strongly supported as the cardinal lesion. The concomitant SGC constitutes a second structural lesion reaching the vicinity of the SSN, but its incremental compressive contribution was minimal in the eyes of the senior operating author (M.J.).

Anatomically, the SSN arises from the superior trunk of the brachial plexus, passes beneath the TSL at the SSNo, and courses posterolaterally through the suprascapular canal beneath the supraspinatus muscle toward the SGNo.^1^ Pathology at the exit site (i.e. SGC) typically produces infraspinatus dysfunction, whereas proximal compromise affects both supraspinatus and infraspinatus muscles. Large cysts may expand proximally to involve the SSNo.

In a cadaveric study of 93 shoulders in 51 cadavers, Wang et al^24^ found that the supraspinatus motor branch arises very close to the SSNo, at a mean absolute distance of 1.5 mm (standard deviation 5 mm) from the TSL. The branching occurred directly as the nerve traversed under the TSL at the notch level in 54% of shoulders, as in our case (Supplementary Video S2, at 0 minute 33 seconds). Al-Redouan et al^1^ found an average canal length of 25.10 mm (standard deviation 3.36 mm) in 30 cadaveric shoulders. In our case, the cyst size was insufficient to span the canal length, supporting the conclusion of another proximal entrapment. Even for the cyst to compress the infraspinatus branch at the SGNo itself, literature shows that size matters. Tung et al^23^ showed that nerve compression occurred more frequently in cases with cyst size greater than 3.1 cm. Rhee et al^21^ suggested the threshold of 2.2 cm to be considered as a criterion for determining the need for decompression surgery, as size more than 2.2 cm was found to be correlated with positive EMG findings for compressive SSN. Yoon et al^25^ performed EMG/NCS only in patients with muscle atrophy of Warner grade >2 on MRI or in those with SGC size >2.5 cm. We found the 1.3 cm cyst in our case barely reaching the nerve, with no frank signs of neural suffering on arthroscopy, and insignificant differential electrophysiologic conduction block. We cannot quantitate the amount of nerve compression the cyst was exerting at the SGNo, but from the macroscopic appearance of the nerve in both notches, we can postulate that most of the entrapment was taking place proximally. This is important to consider, as misjudgment before surgery could have yielded to missing the main entrapment site, especially that neurologically asymptomatic SGCs with spontaneous resolution have been described in literature.^25^

A comparable report of concomitant TSL release and SLAP repair in a professional handball female player has been published with similar clinical findings and successful recovery.^9^ Unlike our case, this professional overhead athlete lacked SGC formation. During arthroscopy, the authors found a type II-SLAP lesion which they repaired and then they shifted to the subacromial space for TSL exposure and release. The TSL was hypertrophic and the SSN had a visible strangulation mark like the nerve in our case (Supplementary Video S2, at 1 min, 58 s). Another report^20^ described the case of a 17-year-old male rower who sustained a large paralabral SGC tracking proximally to the SSNo, resulting in both infra- and supraspinatus weakness. The authors decompressed the cyst intraarticularly, repaired the labrum and then shifted to the subacromial space to decompress the nerve at the TSL. This case differed from ours by the fact that a single lesion produced compression at the 2 notches. This was clear and could not have been overlooked preoperatively as the authors included an MRI section which shows the SGC reaching and occupying the SSNo.

An unusual feature of the present case was the abrupt onset of neuropathy following non-shoulder surgery. The patient denied shoulder symptoms before the meningioma excision but awoke from that surgery with burning posterolateral arm pain and inability to elevate the arm. Pain resolved within weeks and became infrequent dysesthesia, while weakness and atrophy progressed. This clinical pattern resembles a case reported by Economides et al,^6^ where SSNeur developed after an appendectomy procedure. In this case, pain resolved after two weeks, while weakness and atrophy continued to progress. A traction mechanism of injury sustained during patient transfer/positioning under general anesthesia may have precipitated SSNeur in these cases. However, it's important to exclude other differentials such as an acute cervical disc hernia or neuralgic amyotrophy (NA). The clinical presentation in our case is inconsistent with NA as the onset of severe weakness (palsy) peaked concomitantly with pain rather than following a distinct prodromal pain phase, which is the typical sequence in NA. Moreover, the patient's pain lacked the characteristic sudden, excruciating, and widespread quality involving multiple dermatomes usually seen in NA. Electrodiagnostic studies demonstrated a focal, isolated SSNeur, whereas NA classically produces multifocal, patchy involvement across the brachial plexus. The presence of increased latency with preserved motor units further supports a mechanical compression mechanism, contrasting with the diffuse axonal loss and normal latency expected in NA. Imaging findings showing denervation confined to the SSN territory, without involvement of adjacent muscles or nerves, also favor a localized compressive etiology. Furthermore, the patient's rapid postoperative strength recovery is incompatible with the prolonged and often incomplete recovery trajectory typical of NA. Finally, the severe entrapment observed intraoperatively explains the neuropathy distal to the entrapment site. Collectively, these features strongly favor mechanical SSN compression over NA.

In their landmark cadaveric study, Laumonerie et al^15^ described 3 sensory branches arising from the SSN (Fig. 3). Based on this model, the dual entrapment in our case was expected to irritate at least one sensory branch and cause pain. Probably this explains the episodes of sudden posterolateral “electric stabs” that the patient described, and which disappeared after decompression. Though, these episodes were very infrequent, and in between the episodes days or weeks would pass without any pain. The quality of this pain is not classical for SSNeur, which produces burning or dull aches in the posterolateral shoulder. The largely painless presentation in our case may be explained by advanced chronic denervation and predominant muscle atrophy, as suggested by prior authors.^3^^,^^6^^,^^12^

**Figure 3:**
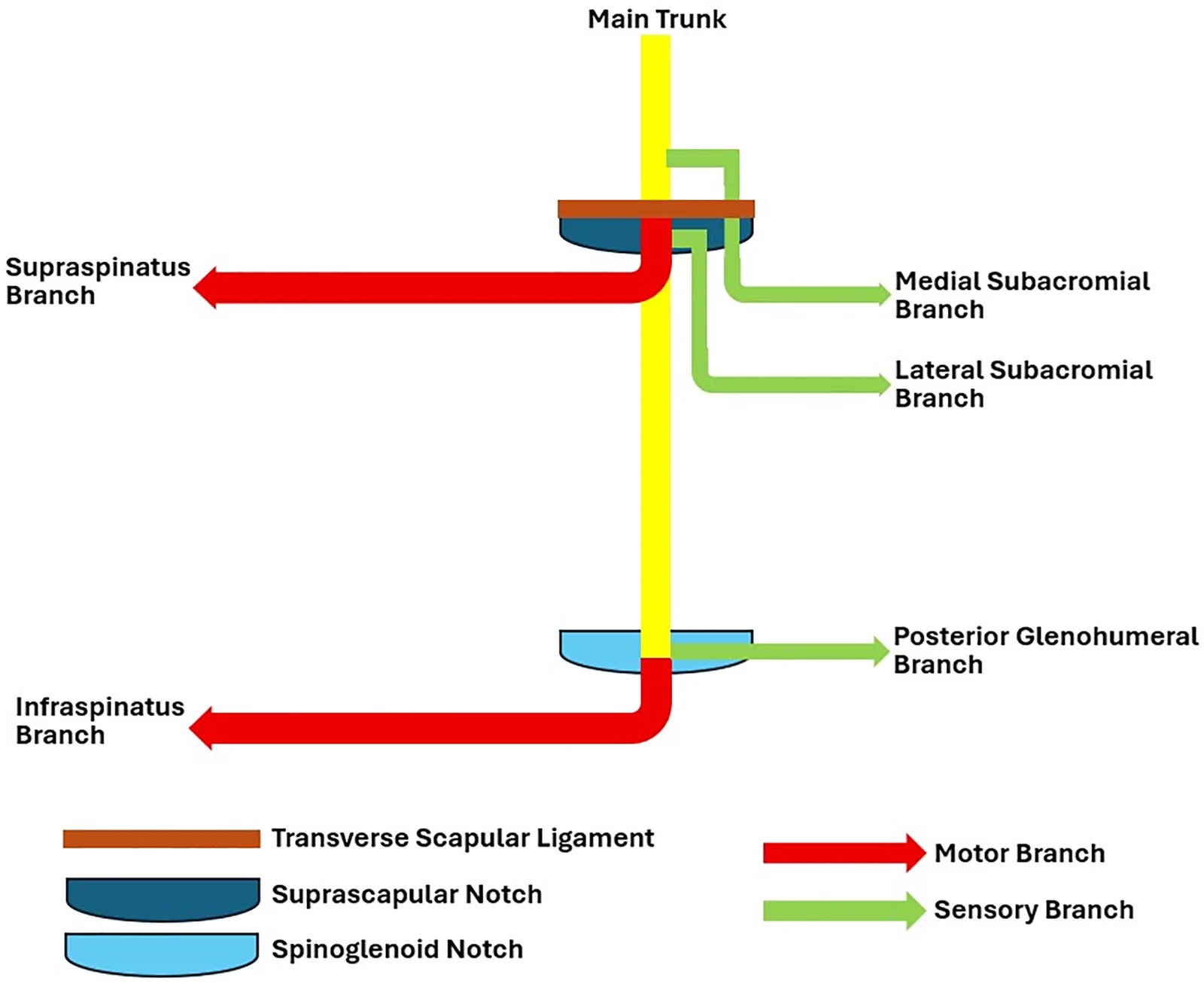
Schematic illustration depicting the most common branching pattern of the suprascapular nerve as compiled from various cadaveric studies.^15^^,^^24^

Surgical decompression of either the SSNo or the SGNo generally yields excellent outcomes. In the largest and newest systematic review on SSN decompression, Sandler et al^22^ pooled data for 730 patients who underwent open/arthroscopic decompression at either notch. Substantial statistically significant improvements were calculated for American Shoulder and Elbow Surgeons (ASES), Constant, Disabilities of the Arm, Shoulder, and Hand (DASH), Subjective Shoulder Value, University of California Los Angeles, and visual analog scale pain scores. The gains in the ASES, Constant, Subjective Shoulder Value, and University of California, Los Angeles scores were 25, 30, 32 and 16 points, respectively. DASH and visual analog scale pain scores reduced by 50 and 4.2 points, respectively. Ninety-eight percent of patients were able to return to sport or military duty with 96% able to return at their previous functional level. Despite that, persistent symptoms were noted among 12% of patients, irrespective of whether nerve decompression was performed at the SSNo or the SGNo. The surprising result was that 32% continued to have some degree of electrodiagnostic testing abnormality. Perhaps we can speculate that persistent neuropathy in few of these cases may reflect uninvestigated, untreated, or missed concomitant entrapment at the other notch.

Arthroscopic decompression at the SSNo was pioneered by Lafosse et al,^14^ with excellent outcomes. They reported statistically significant improvements in pain (from 7/15 preoperatively to 14.5/15 postoperatively, *P* < .001) and Constant Murley scores (from 60.3 preoperatively to 83.4 postoperatively, *P* < .001) at 15-month follow-up.^14^ In a recent series, Krishnan et al^13^ reported good to excellent results following arthroscopic SSN decompression at the SGNo, with all 8 patients showing significant improvements in both ASES (61.5 preoperatively to 90.3 at 1-year follow-up) and Constant Murley scores (66.8 preoperatively to 93.4 at 1-year follow-up). While it is beyond the scope of this report to discuss intra-articular vs. subacromial techniques for SGC decompression, the subacromial approach may offer an added advantage of direct visualization of the cyst wall and the SSN, while avoiding excessive medial capsular dissection through the glenohumeral joint and its attendant risk of iatrogenic nerve injury.^7^ Ghodadra et al^7^ described a technique of concomitant arthroscopic decompression of the SSN at the SGNo and SSNo through the subacromial space, but no long term clinical outcomes are available yet. The senior operating surgeon (M.J.) in the current report favors intra-articular decompression of SGCs as this disrupts the one-way valve mechanism responsible for accumulating cyst fluid; this approach has been highly successful in his other cases. However, he elected to expose the cyst from above in this specific case because of tactical preference. SSNo exposure requires medial bursectomy and clearing up of the perimuscular fat around the supraspinatus muscle. Extending the bursectomy a little posteriorly (to the scapular spine) exposes the raphe between supraspinatus infraspinatus and facilitates the identification of the cyst underneath.

Limitations of this report include the lack of follow-up imaging and electromyography as the patient relocated abroad after surgery. However, ongoing clinical follow-up was maintained remotely, including video-based confirmation of postoperative strength recovery and the completion of 2 postoperative patient-reported outcome questionnaires by email. Another limitation is that strength measurements were obtained with a non-calibrated handheld dynamometer. However, the unaffected contralateral shoulder served as an internal control, and the substantial preoperative strength deficit (80%) and the near-complete postoperative recovery ameliorates the qualitative effect of uncalibrated strength measurement.

## Conclusion

Dual-notch suprascapular nerve pathology can occur when a SGC coexists with an independent proximal entrapment at the SSNo. In SGC patients with disproportionate involvement of the supraspinatus on clinical examination, electrodiagnostic studies, or on imaging, a smaller-than-expected cyst that does not extend superiorly into the supraspinatus notch should prompt evaluation for an additional proximal lesion.

According to our institutional policy, single-patient case reports do not require IRB approval or formal ethical committee review. Written informed consent was obtained from the patient for publication of this case report and any accompanying images or videos.

## Disclaimers

Funding: The authors did not receive any outside funding to conduct this study.

Conflicts of interest: The authors, their immediate families, and any research foundation with which they are affiliated have not received any financial payments or other benefits from any commercial entity related to the subject of this article.

Patient consent: Written informed consent was obtained from the patient for publication of this case report and accompanying images and videos. Patient identity was deidentified throughout the entire work.

## Supplementary Data

Supplementary Video A different case with no suprascapular neuropathy demonstrating normal suprascapular nerve at the suprascapular notch.

Supplementary Video S1 Part of the preoperative clinical examination.

Supplementary Video S2 Operative procedure and intraoperative findings.

Supplementary Video S3 Postoperative patient-demonstrated strength.
